# Retraction: Polymorphism of 9p21.3 Locus Is Associated with 5-Year Survival in High-Risk Patients with Myocardial Infarction

**DOI:** 10.1371/journal.pone.0095513

**Published:** 2014-04-11

**Authors:** Anna Szpakowicz, Witold Pepinski, Ewa Waszkiewicz, Dominika Maciorkowska, Małgorzata Skawronska, Anna Niemcunowicz-Janica, Robert Milewski, Sławomir Dobrzycki, Włodzimierz Jerzy Musial, Karol Adam Kaminsk

The authors and the editors retract this publication.

The authors have identified a mistake in the genotyping analyses underlying this study. Although the result that a 9p21.3 polymorphism affects the prognosis after STEMI in high-risk patients stands, the re-evaluation of the data shows that the allele A is the correct risk allele for death for the rs4977574 polymorphism, and not allele G as reported in the article.

The authors have completed additional work to validate the genotyping results from this study and these data will be published as a new publication.

The authors and the editors apologize to the readers.
